# Supplementary material for: Patterns of Intron Gain and Loss in Fungi
Source: PLoS Biol. 2004 Nov 30;2(12):e422. doi: 10.1371/journal.pbio.0020422 (PMC532390; doi:10.1371/journal.pbio.0020422)
Supplement: Table S1 — Also available at http://genes.mit.edu/NielsenEtAl/. (4.3 MB ZIP). [file pbio.0020422.st001.zip › NielsenEtAl/html/661.html]

AN6209.1.NCU06187.1.MG03645.1.FG09185.1


```
 CLUSTAL W (1.82) Multiple Sequence Alignments - Introns Inserted


Sequence 1: NCU06187.1	482 aa
Sequence 2: MG03645.1	507 aa
Sequence 3: AN6209.1	489 aa
Sequence 4: FG09185.1	485 aa
Alignment Length: 514 aa
Number Identitical Residues: 208 aa
Alignment Score (without introns) 11089


MG03645.1 	-MPDYDSYQ~TPLGMRYAS~REMLTLFSPRSRNNTWRQLWIHLAEAEHE~LGLPQVTQEK
NCU06187.1	-MSSYDKYQ0TPLATRYAS~QEMMSIFSARTRASTWRQLWVWLAEAEKE~LGLN-IPDEA
FG09185.1 	-MAAYDTYQ~TSLTGRYCS~QELSHLFSQRSRHSTWRKLWLYLAESEKE~LGIPTITDEA
AN6209.1  	MSSSNDVYQ~TPLNSRYAS1DEMKYLFSPRNRFSTWRQLWLWLAEAEKE1LGLP-ITDEA
          	  .  * ** *.*  **.*  *:  :** *.* .***:**: ***:*:* **:  :.:* 

MG03645.1 	IDELKAHAHVTDEAFEVIKAEEKIRR~H~DVMA~AVHAYGKEAPGAAGIIHLG~ATSCYV
NCU06187.1	IQQMREHVTVSDKAFDVAREYENKFR0H~DVMA~HVHAYGEDAPAAAGHIHLG0ATSCYV
FG09185.1 	LEQMRANLVVTDDDFETARVEEKIRR~H0DVMA0HVHAFGQVAPAAAGIIHYG~ATSCFV
AN6209.1  	IEQMRKHTTINDEEFKVAAEEEKRRR~H~DVMA~HVHAYGQVAPAAAGIIHWG~ATSCYC
          	::::: :  :.*. *..    *:  * * ****  ***:*: **.*** ** * ****: 

MG03645.1 	~TDNAELIFIKKGLELLLPKLAKVISNLQKFALKYKDLPTLGYTHLQPAQLITVGKRVCQ
NCU06187.1	1TDNADLIFIQKALDLILPKLAKVIQNLKEFALKYKDTPTLGFTHYQPAQLITIGKRACQ
FG09185.1 	~TDNTELILMRDALDLLIPKLAKVLSNLQSFALEWKNEPTLSFTHLQPAQISTVGKRAAA
AN6209.1  	~TDNADLIFLRDGLNILIPKLAVVIDKLSAFAKQYKDLPCLGFTHGQPAQLVTVGKRACL
          	 ***::**:::..*::::**** *:.:*. ** ::*: * *.:** ****: *:***.. 

MG03645.1 	WIQELTMDLEDIEHVLSALKLRGIQGTTGTKASFMELFQGDEVKVNKLNDILCKKLGFQG
NCU06187.1	WIQELVMDLEDIETVRERLQFRGAQGTTGSQATFLELFEGDADKIVELNKILCKKAGFPS
FG09185.1 	WAQDLLMDLNEFERVRADLKFRGAQGTTGTQASFLEIFAGDHDKCDKLNELLCQKAGFEE
AN6209.1  	WIQDLLMDLRNLERARDDLRFRGVKGTTGTQASFLQIFNGDHDKVEQLDELVTKKAGFDS
          	* *:* ***.::* .   *::** :****::*:*:::* **  *  :*:.:: :* **  

MG03645.1 	VYDISTQTYTRKVDLRVANALSAFGATAERIGLDIRHLANWKEIEEPFEAGQIGSSAM~A
NCU06187.1	TYPISTQTYTRKVDLRVANAVCAIGATAERIASDIRHLANLKEMEEPFEKSQIGSSAM~A
FG09185.1 	CYDISTQTYTRKVDCLVANAVTGFGTSVTKIASDLRHLATMKEVGEPREKGQIGSSAM0A
AN6209.1  	AFIISSQTYSRKIDVDVANALGSFGSTCERIGIDIRHLAMLKEVEEPFEKDQIGSSAM0A
          	 : **:***:**:*  ****: .:*::  :*. *:****  **: ** * .******* *

MG03645.1 	YKVSISAHRHTFADGRSANIARLQRNPMRSERITSLGRKLSTLSVNFTKTFEVQMMERTL
NCU06187.1	YK----------------------RNPMRSERIAALSRKLANLPVNFSNTFKAQWFERTL
FG09185.1 	YK----------------------QNPMRSERIASLARVLQGKAANFQSTHSTQWMERSL
AN6209.1  	YK----------------------RNPMRSERLCSLGRHLQNLPKDALDTYSAQWFERSL
          	**                      :*******: :*.* *   . :  .*...* :**:*

MG03645.1 	~------DDSAI~RRIDIPEMFLLADAILISLD~NVTDGLVVYPATVRSRVMQELPFMAT
NCU06187.1	~------DDSAI~RRMDIPEMFLLADSILLALD~NVTNGLVVYPSVVRAHIDQELPFMAT
FG09185.1 	~------DDSAC0RRMDIPEMFLLADAIAITLQ~NVTEGLVVFPLKIHSNIMAELPFMIT
AN6209.1  	0LLMADKDDSAI~RRISIPELYLCADACLILLN1NVTSGFVVYPEVIKRRVNDELPFMAT
          	    :..****  **:.***::* **:  : *: ***.*:**:*  :: .:  ***** *

MG03645.1 	~EN~IIMKMVAQGASRQE~CHEEIR~VLSHQASHAVKMEGKSNDLLERIKNTEYFKPVHA
NCU06187.1	~ES~ILMKLAQHGVSRQD~AHEEIR~VLSHQASAVVKQEGGKNDLLERIKKSEFFKPVWG
FG09185.1 	~EN~VIMRLVAMGVSRQE2AHEQIR~VLSFEASHQVQSLGKPNDMVERIKKTDFFKPIWA
AN6209.1  	2EN0VIMACVKKGLSRQD~AHEEIR1VLSHQASDNVKKHGKNNDLLDRIRRTEFFAPILG
          	 *. ::*  .  * ***: .**:** ***.:**  *:  *  **:::**:.:::* *: .

MG03645.1 	ELDAMLDPALFTGCSQSIVERYCGPDGTVEKALAPYKEHIASAATAELSV
NCU06187.1	EIDGLVDPALFIGNCPRIVEDYCN--GQVAEKLSRYQETLSKAETAQLSI
FG09185.1 	DLDDMMKPELYIGRSAQLVDKFCGPGGKLEKKLQPYQEVIQKAKAAELNV
AN6209.1  	ELDALLDPSTFVGRAPQQVEKFTS--TEVKKALQPYETYLATAETSALHV
          	::* ::.*  : * .   *: : .    : : *  *:  : .* :: * :
```
